# Neuronal Cell-Intrinsic Defects in Mouse Models of Down Syndrome

**DOI:** 10.3389/fnins.2019.01081

**Published:** 2019-10-10

**Authors:** Alessandra Maria Adelaide Chiotto, Martina Migliorero, Gianmarco Pallavicini, Federico Tommaso Bianchi, Marta Gai, Ferdinando Di Cunto, Gaia Elena Berto

**Affiliations:** ^1^Neuroscience Institute Cavalieri Ottolenghi, University of Turin, Turin, Italy; ^2^Department of Molecular Biotechnology and Health Sciences, University of Turin, Turin, Italy; ^3^Department of Neuroscience, University of Turin, Turin, Italy

**Keywords:** Down syndrome, neural differentiation, Ts65Dn, Ts2Cje, dendritic spines

## Abstract

Down Syndrome (DS) is the most common genetic disorder associated with intellectual disability (ID). Excitatory neurons of DS patients and mouse models show decreased size of dendritic field and reduction of spine density. Whether these defects are caused by cell autonomous alterations or by abnormal multicellular circuitry is still unknown. In this work, we explored this issue by culturing cortical neurons obtained from two mouse models of DS: the widely used Ts65Dn and the less characterized Ts2Cje. We observed that, in the *in vitro* conditions, axon specification and elongation, as well as dendritogenesis, take place without evident abnormalities, indicating that the initial phases of neuronal differentiation do not suffer from the presence of an imbalanced genetic dosage. Conversely, our analysis highlighted differences between trisomic and euploid neurons in terms of reduction of spine density, in accordance with *in vivo* data obtained by other groups, proposing the presence of a cell-intrinsic malfunction. This work suggests that the characteristic morphological defects of DS neurons are likely to be caused by the possible combination of cell-intrinsic defects together with cell-extrinsic cues. Additionally, our data support the possibility of using the more sustainable line Ts2Cje as a standard model for the study of DS.

## Introduction

Trisomy for human chromosome 21 (HSA21) causes Down syndrome (DS) in one every 800 live births (de Graaf et al., 2017), making it the most common genetic cause of developmental delay and intellectual disability (ID). DS is characterized by several phenotypes affecting many organ systems, including CNS abnormalities that lead to cognitive and motor impairment, congenital heart defects, megakaryocytic leukemia and early onset Alzheimer’s disease (AD) (Haydar and Reeves, 2012; Hibaoui et al., 2014; Hartley et al., 2015). Thanks to the constant improvement of medical care and to increased access to it, a vast majority of these problems can be now addressed medically (e.g., megakaryocytic leukemia) or surgically (e.g., congenital heart defects). However, despite the presence of several medical trials, cognitive impairment remains a limiting factor in DS patients, by reducing the accomplishment of personal and social goals.

A number of studies performed on patients and animal models demonstrated that the brain structures more affected in DS are the hippocampus, the cerebellum and the cerebral neocortex (Dierssen et al., 2009; Rueda et al., 2012). MRI studies revealed that neuro-anatomic abnormalities in the cerebral cortex are correlated with the cognitive profile of DS patients (Raz et al., 1995; Pinter et al., 2001; Lott, 2012). However, the alterations that characterize neocortical structure and its development in DS patients are less studied than the abnormalities of other districts. Qualitative and quantitative defects, such as reduction of dendritic arborizations, decreased synaptic contacts and altered information processing, have been documented in DS patients cortical neurons (Wisniewski et al., 1984; Golden and Hyman, 1994).

Cortical alterations similar to those found in patients have also been described in Ts65Dn mice (Dierssen et al., 2003) the most commonly used and best characterized rodent model of DS (Davisson et al., 1993). Ts65Dn mice possess an extra chromosome, containing approximately two-thirds of HSA21 orthologous genes. Recently, Ts[Rb (12.17^16^)]2Cje (Ts2Cje) mice have been established, after a fortuitous translocation of the Ts65Dn extra chromosome to chromosome 12 (Villar et al., 2005). Ts2Cje possess the same amount of triplicated DNA sequence as Ts65Dn, but the stable rearrangement spares fertility in males and increases the frequency of transmission of the segmental trisomy through the female germline. These differences make Ts2Cje mice a much easier model to study, as compared to Ts65Dn animals.

Since the presence of an extra, freely segregating chromosome may contribute to DS phenotypes, Ts65Dn mice may represent a more faithful model. However, it is noteworthy that possessing an entire extra HSA21 is not necessary for DS-related ID, since a subset of DS patients show partial trisomy, associated with chromosome 21 translocation and fusion events. Ts2Cje mice have not been characterized as deeply as the Ts65Dn line, but they show some of the DS-relevant phenotypes previously found in Ts65Dn mice, such as structural dendritic spine abnormalities, ventriculomegaly and altered neurogenesis (Villar et al., 2005; Ishihara et al., 2010; Raveau et al., 2017). Considering the difficulties of breeding the Ts65Dn line, a deeper characterization of Ts2Cje mice could provide valuable insight for further establishing this more tractable model. In addition, in both models, it is not well understood whether cortical neuron abnormalities are primary and cell-autonomous or the result of altered dynamics of neurogenesis.

To address these issues, we cultured cortical neurons from newborn Ts65Dn and Ts2Cje mice and evaluated their ability to differentiate in *in vitro* conditions.

Our data indicate that, in both mouse models, axonogenesis and dendritogenesis are unaffected, while dendritic spines are both reduced and immature, suggesting that only the latter phenotypes are a cell-autonomous consequence of the genetic imbalance.

## Materials and Methods

### Mice

Ts65Dn and Ts2Cje lines were bred accordingly to Jackson’s Laboratories directions, conforming to the Italian laws on animal experimentation and under the supervision of the veterinary service of our animal facility. Mice were genotyped with PCR using primers spanning the translocation site.

### Neuronal Primary Cell Culture and Transfection

Mouse cortical neurons were isolated from Ts65Dn and Ts2Cje pups and euploid litters on the day of birth (P0) as previously described (Beaudoin et al., 2012). Briefly, PCR was performed on a small amount of tissue obtained from the tail and mice with the same genotype were then processed as a single individual. Brains from both euploid and trisomic mice were extracted from the skull, meninges were removed, the two hemispheres were separated, hippocampus removed, cortices were isolated and transferred into 1 ml of pre-warmed 2,5% trypsin (Sigma) for 15 min at 37°C. Cortices were then washed five times with HBSS (Thermo Fisher), DNAseI (Promega) was added to the last wash and incubated at 37°C for 10 min. Subsequently, cells were carefully disaggregated with a P1000 sterile filtered tip eight to ten times, counted and plated in Mem Horse medium (MEM 1×, 10% horse serum, 2 Mm L-glutamine) on poly-L-lysine (Sigma, 1 mg/ml.) pre-coated coverslips with a density of 32,500 cells/cm^∧^2. After 4 h, medium was changed into Neurobasal (Thermo Fisher) supplemented with 2% B27 (Thermo Fisher) and 2 mM L-glutamine (Gibco). Fresh supplemented Neurobasal was added to cultures every 4 days after the removal of half of the medium.

To highlight neuronal morphology for dendritogenesis and dendritic spines analysis, pEGFP-C1 plasmid (Clontech) was transfected using Lipofectamine LTX (Thermo Fisher) according to manufacturer’s indications.

### Immunofluorescence, Image Acquisition, and Analysis

Neurons were fixed with 4% paraformaldehyde in PBS for 10 min, quenched with 50 mM NH_4_Cl for 15 min, permeabilized with 0.1% Triton X-100/PBS for 5 min. Non-specific sites were blocked with 5% BSA/PBS for 30 min. Immunofluorescence (IF) was performed using the anti-GFP antibodies (Rabbit polyclonal AB290, 1:1000, Abcam), followed by incubation with appropriate Alexa Fluor-conjugated secondary antibodies (Molecular Probes). Polymeric F-actin was detected with Tritc or Fitc phalloidin (Sigma). Interneurons were identified with GAD67 staining (mouse monoclonal, 1:100, Abcam). Axons were stained with anti neurofilament H (mouse monoclonal SMI 32, 1:200, Biolegend) and pre-synaptic sites were stained with Bassoon (mouse monoclonal, 1:200, Stressgene).

Images were acquired with ViCo (Nikon) fluorescent microscope or with SP5 Leica confocal microscope. All analyses were performed with FiJi software (Schindelin et al., 2012). Traces of neurites were obtained using the NeuronJ plugin for FiJi. In brief, Z-stacks of GFP transfected neurons were projected on one plane (“maximum projection”) and traces were manually drawn with a line. Concentric circles were centered on cell soma and the number of intersections was counted manually. Total dendritic length was measured with FiJi “segmented line” tool. Dendritic spines were counted manually on 10 μm dendritic segments, 20 μm far from cell soma. At least two segments per cell were analyzed.

## Results

### Neuronal Polarity Is Not Altered in Ts65Dn and Ts2Cje Mouse Cortical Neurons

**FIGURE 1:**
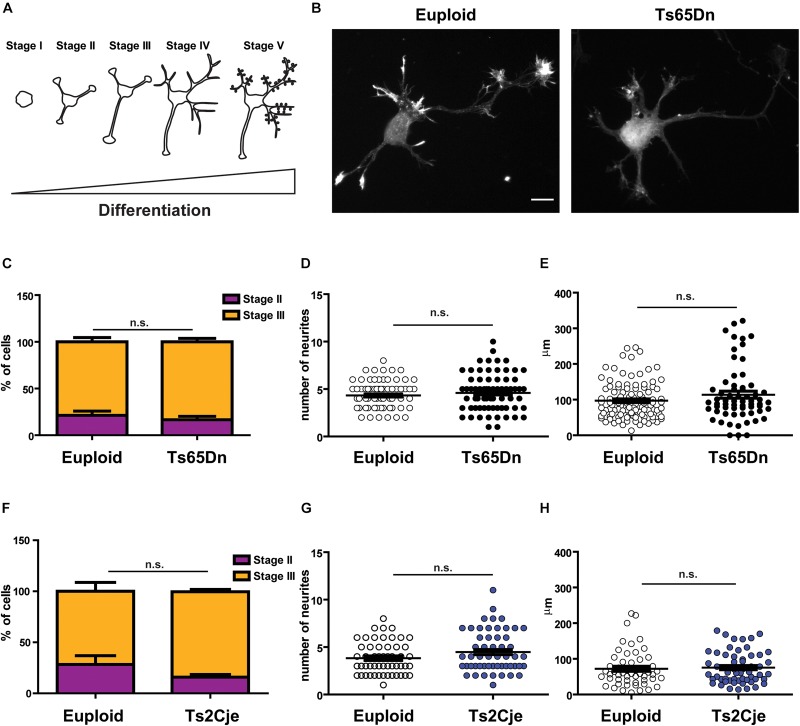
Neuronal polarity is not altered in Ts65Dn and Ts2Cje mouse cortical neurons. **(A)** Schematic representation of neuronal differentiation. We observed that during the first 3 days in culture, neurons generate and specify the axon (stages I to III). Next, around day in culture (DIV) 7, cells start to elongate dendrites (stage IV) and after 2 weeks they begin to be synaptically active (stage V). **(B)** Euploid (left panel) and Ts65Dn (right panel) cortical neurons at DIV3, stained for filamentous actin (phalloidin). Cells appear properly differentiated and show no evidence of delay or maturation alteration. A similar pattern was observed in Ts2Cje cells. **(C,F)** Percentage of cells in stage II or stage III after 3DIV (**C:** euploid in stage II *n* = 44, stage III *n* = 144, from 5 mice; Ts65Dn in stage II *n* = 18, stage III *n* = 94, from 4 mice. **F:** euploid in stage II *n* = 15, stage III *n* = 38, from 4 mice; Ts2Cje in stage II *n* = 10, stage III *n* = 51, from 4 mice). Euploid vs. Ts65Dn *p* = 0.32 (stage II) and *p* = 0.65 (stage III); euploid vs. Ts2Cje *p* = 0.28 (stage II) and *p* = 0.25 (stage III). **(D,G)** Total number of primary neurites emerging directly from cell soma (**D:** 78 cells from 5 mice for euploid, 75 cells from 4 mice for Ts65Dn; 1G: 53 cells from 6 mice for euploid, 61 cells from 6 mice for Ts2Cje). Euploid vs. Ts65Dn *p* = 0.32; euploid vs. Ts2Cje *p* = 0.07. **(E,H)** Axon length after 3 DIV. All the evaluated parameters showed no significant differences in the two trisomic models, when compared to the matched euploid controls (**E:** 105 cells from 5 mice for euploid, 63 cells from 4 mice for Ts65Dn; **H:** 53 cells from 6 mice for euploid, 57 cells from 6 mice for Ts2Cje). Euploid vs. Ts65Dn *p* = 0.09; euploid vs. Ts2Cje *p* = 0.71. Statistics: Unpaired two tailed Student’s *t*-test. *P* > 0.05 was considered not significant. Error bars represent SEM. Scale bar = 10 μm.

To evaluate whether the cortical defects described in DS patients and in mouse models could be attributed to cell intrinsic defects, we resorted to the use of *in vitro* primary cultures, obtained from post-natal 0 (P0) pups. Neuronal cultures were prepared from both Ts65Dn and Ts2Cje mice (Villar et al., 2005). In both cases, euploid littermates were used as matched controls. Cultures were composed by a majority of excitatory neurons. Indeed, they contained approximately 20% inhibitory neurons, with no significant differences between genotypes (Supplementary Figure 1A). Moreover, we only analyzed cultures containing less than 20% glial cells (Supplementary Figure 1C)(see methods for technical details about the procedure). We evaluated all the main stages of neuronal development (Banker and Goslin, 1988): axonogenesis (days *in vitro*∼DIV-3), dendritogenesis (∼DIV7) and synaptic maturation (∼DIV14) (Figure 1A). Three days after plating, we counted the percentage of cells in stage II (multipolar cells, with equally long neurites) and in stage III (polarized SMI^+^ cells, Supplementary Figure 1B) (Banker and Goslin, 1988): we did not observe any difference in the distribution of cells between the two stages in the two analyzed strains (Figures 1B,C,F). It has previously been reported that the number of projections is higher and axonal length is increased in cultured hippocampal neurons obtained from Ts65Dn and in cortical neurons obtained from patients’ samples (Sosa et al., 2014). To evaluate whether cortical neurons from Ts65Dn and Ts2Cje would behave the same, we counted the number of primary neurites emerging directly from the cell soma and the length of the axon after 3 days in culture. In both models, we did not appreciate any alteration in the number of primary projections (Figures 1D,G). In addition, although in both genotypes the average axonal length tended to a slight increase, the differences from controls were not statistically significant (Figures 1E,H). Together, these data indicate that cortical neurons in primary culture, obtained from post-natal brain of two different mouse models of DS, show a normal pattern of neuritogenesis, with no significant alterations in the stage progression or in the axonal outgrowth.

### Dendritic Arborization Is Unaffected in Ts65Dn and Ts2Cje Cortical Neurons

**FIGURE 2:**
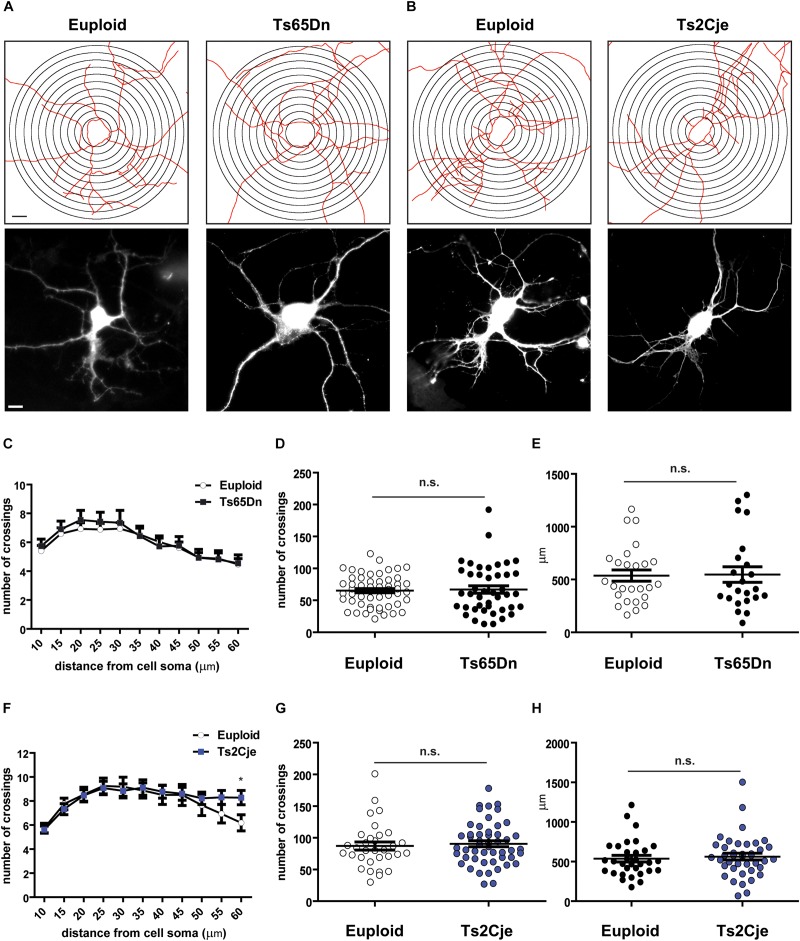
Dendritic arborization is unaffected in Ts65Dn and Ts2Cje cortical neurons. **(A,B)** Skeletonization with Sholl analysis target superimposition (upper panels) and GFP fluorescent images (lower panels) of DIV7 cortical neurons. **(C,F)** Sholl analysis of cells images processed as in panels **(A,B)** (**C:** 55 cells from 8 mice for euploid, 43 cells from 5 mice for Ts65Dn; **F:** 32 cells from 4 mice for euploid, 50 cells from 6 mice for Ts2Cje). In panel **F**, ^∗^*p* = 0.03. **(D,G)** Total number of crossings counted in panels **(C,F)**, respectively. Euploid vs. Ts65Dn *p* = 0.78; Euploid vs. Ts2Cje *p* = 0.65. **(E,H)** Total dendritic length measured within the Sholl area (**E:** 26 cells from 8 mice for euploid, 23 cells from 5 mice for Ts65Dn; **H:** 32 cells from 4 mice for euploid, 40 cells from 6 mice for Ts2Cje). Euploid vs. Ts65Dn *p* = 0.90; euploid vs. Ts2Cje *p* = 0.60. Statistics: unpaired two tailed Student’s *t*-test. *P* > 0.05 was considered not significant. Error bars represent SEM. Scale bar is 10 μm.

During neuronal differentiation, the establishment of neuronal polarity and axon sprouting are followed by rapid growth of the minor neurites into dendrites. *In vitro*, this step takes place around DIV7 (Takano et al., 2015). Previous *in vivo* work has reported that Ts65Dn layer III pyramidal neurons display simplified branching pattern and shorter dendritic length (Dierssen et al., 2003), while no data are currently available for Ts2Cje. We thus evaluated the differentiation potential of Ts65Dn and Ts2Cje cortical neurons, by analyzing their capability to form dendrites after 7 days *in vitro*. To clearly highlight the dendritic tree, neurons were transfected at DIV5 with a GFP expressing plasmid. Sholl analysis (Sholl, 1953) was performed on GFP-positive cells (Figures 2A,B) counting the number of intersections up to a radius of 60 μm from cell center. We did not analyze larger radii, because of the high number of breaks taking place at the tiny tip of dendrites. Interestingly, this analysis did not reveal significant changes in the number of intersections (Figures 2C,F). Even the cumulative number of intersections (Figures 2D,G), as well as the total dendritic length (calculated inside the Sholl’ area) (Figures 2E,H) were not different from controls in both genotypes.

**FIGURE 3:**
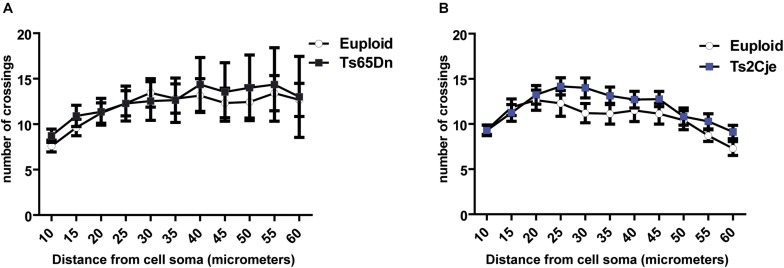
Dendritic arborization is not altered in Ts65Dn and Ts2Cje neurons at later stages of maturation. **(A,B)** Sholl analysis of DIV14 GFP + neurons from both Ts65Dn and Ts2Cje (and age matched controls). The number of intersections between euploid and trisomic neurons is comparable also at this time point (**A:** 27 cells from 3 mice for euploid, 11 cells from 3 mice for Ts65Dn; **B:** 15 cells from 3 mice for euploid, 16 cells from 3 mice for Ts2Cje). Euploid vs. Ts65Dn *p* ≥ 0.5 for all the points; euploid vs. Ts2Cje *p* ≥ 0.5 for all the points. Statistics: unpaired two tailed Student’s *t*-test. Error bars represent SEM. *P* > 0.05 was considered not significant.

Considering the previous work showing that Ts65Dn cortical neurons display dendritic alterations *in vivo* (Dierssen et al., 2003), we next asked whether these defects may depend on the inability of neurons to properly maintain, rather than establish, the structure of dendritic fields. Indeed, around DIV7-9, differentiating mouse neurons display a dynamic phase characterized by branching instability, with continuous progression and retraction of the neurites. This is followed, around DIV10-15, by a stabilization phase, leading to the final dendritic configuration (Baj et al., 2014). To evaluate whether the stabilization phase is affected in trisomic mice, we performed Sholl analysis also in mature Ts65Dn and Ts2Cje neurons, at DIV14. As expected, the number of intersections is increased at this time, as compared to DIV7. However, even at this stage, no differences were detected between trisomic and euploid neurons (Figures 3A,B). These data suggest that cortical neurons of trisomic mice, grown in *in vitro* conditions, possess a similar intrinsic capability to generate dendrites and to establish a dendritic arbor as euploid controls.

### Dendritic Spines of Trisomic Neurons in Primary Culture Show Immature Morphology and Reduced Density

**FIGURE 4:**
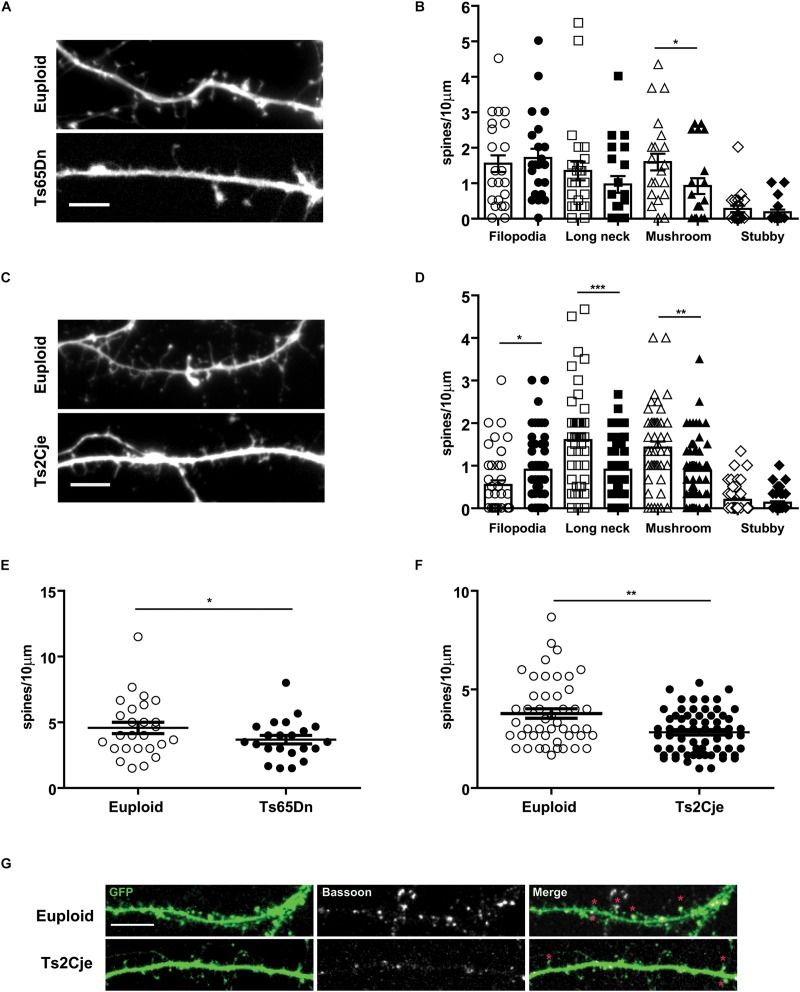
Spine morphology is altered in Ts65Dn and Ts2Cje cortical neurons. **(A,C)** High magnification images of dendrites of euploid and trisomic DIV14 cortical neurons (respectively, Ts65Dn and Ts2Cje); scale bar 5 μm. **(B,D)** Quantification of different classes of spines calculated as the density of spines on ten micrometers. Based upon morphological parameters, spines were classified as filopodia, long neck, mushroom, stubby. In Ts65Dn and Ts2Cje the number of long neck and mushroom spines was decreased. The amount of filopodia spines was significantly higher only in Ts2Cje. White symbols represent euploid mice, black ones Ts65Dn in panel **(B)** and Ts2Cje in panel **(D)**. Bars represent mean. Error bar is SEM. **(E,F)** Quantification of total number of spines calculated as the density of spines on ten micrometers segments. In trisomic conditions the total number of spines is decreased. (**B–E**: 26 cells from 3 mice for euploid, 22 cells from 3 mice for Ts65Dn. **D–F**: 47 cells from 5 mice from euploid, 64 cells from 6 mice from Ts2Cje). **B**-Euploid vs. Ts65Dn (each group *p*-value in the same order represented in the graph): *p* = 0.62, *p* = 0.22, ^∗^*p* = 0.03, *p* = 0.26. **D**-Euploid vs. Ts2Cje: ^∗^*p* = 0.013, ^∗∗∗^*p* = 0.0004, ^∗∗^*p* = 0.0018, *p* = 0.41. **E:** ^∗^*p* = 0.04. **F:** ^∗∗^*p* = 0.0024. Statistics: Two-tailed Mann–Whitney test. Error bars represent SEM. *P* > 0.05 was considered not significant. **(G)** Representative images of spine contacts. Stars indicate GFP (filling post-synaptic protrusions) and Bassoon (pre-synaptic marker) positive excitatory spines in the two genotypes. Error bars represent SEM.

We next wondered whether Ts65Dn and Ts2Cje neurons in culture show alterations of dendritic spines. Indeed, *in vivo* studies have reported that Ts65Dn neurons exhibit a reduced number of spines, which are further characterized by immature shape and function (Dierssen et al., 2003; Belichenko et al., 2004). To further investigate this topic, we transfected at DIV5 Ts65Dn, Ts2Cje and matched control neurons, with a GFP expressing plasmid. We then quantified the density of dendritic spines at DIV14. Spines were grouped in four classes, as previously described with morphological parameters (Harris et al., 1992): filopodia (without a visible head), stubby (no distinguishable neck), long neck and mushroom (defined as active contacts, Figure 4G). Concerning Ts65Dn, we observed a slight decrease of the long neck spines and a significant decrease of mushroom spines. No dramatic changes were seen in stubby and filopodial spines (Figures 4A,B). The overall spine density (spines/10 μm segment) was also decreased in Ts65Dn, compared to euploid cells (Figure 4E). In Ts2Cje neurons, we observed a similar alteration of spine phenotype as the one observed in Ts65Dn. In Ts2Cje the difference between control and trisomic neurons was significant for all morphological classes, with the only exception of stubby spines (Figures 4C,D). In addition, also in Ts2Cje, spine density was significantly reduced with respect to controls (Figure 4F).

Taken together, these data indicate that Ts65Dn and Ts2Cje neurons have an intrinsically compromised capability to form and mature dendritic spines.

## Discussion

Down syndrome is a genetic disorder characterized by a large cohort of symptoms that greatly vary in both penetrance and severity. Nevertheless, all DS patients share the common hallmark of ID (Chapman and Hesketh, 2000). Much information is available about the abnormalities existing in hippocampus and cerebellum (Dierssen et al., 2009; Rueda et al., 2012). Much less is known about the alterations produced by HSA21 trisomy on cortical circuits, which are likely to significantly contribute to ID (Raz et al., 1995; Pinter et al., 2001; Lott, 2012). In this work, we investigated more in depth the possible cellular basis of cortical structure alterations that characterize DS brain. We evaluated the ability of cortical neurons obtained from Ts65Dn and Ts2Cje to complete the differentiation program within the minimal environment of 2D culture condition. In both models, we could not detect significant impairment or delay in establishment of neuronal polarity, axon outgrowth and dendritogenesis. In particular, the normal axonal development is in accordance with data obtained with transplantation of human DS neurons, differentiated from induced pluripotent stem cells, into adult mice brain (Real et al., 2018). In this study, chronic *in vivo* imaging revealed that DS neurons had a normal pattern of axon development, with rates of both axon growth and retraction similar to those of control neurons. On the contrary, our data are in partial contrast with previous work, in which an increased length of the axon in Ts65Dn cultures was described (Sosa et al., 2014). However, the two experimental conditions differ for cellular type (cortical vs. hippocampal neurons), culturing substrate (poly-L-lysine vs. laminin) and timing of axonal length measurement (72 h vs. 24 h).

In the case of dendritogenesis, we were not able to detect significant differences between the two genotypes and their matched littermate controls in dendritic tree development, complexity and total length.

Data about dendritic arborization in DS mouse models and humans are quite heterogeneous. For instance, in one study (Haas et al., 2013) dendritic fields in Ts1Rhr and Tc1 mice were relatively normal. In contrast, studies performed on both DS patients (Becker et al., 1991) and Ts65Dn mice (Dierssen et al., 2003) reported a simplification of dendrites in cortical neurons. However, age specific differences could exist. Analyses performed in infants with DS (< 6 months of age) indicated a higher number of intersections, particularly evident in cortical layer III cells (Becker et al., 1991). Later on, already after 6 months of age, the reverse situation was found, with reduced dendritic arborization in DS individuals respect to healthy age matched controls (Becker et al., 1991). These results indicate that in DS there is a dramatic cessation of the neuronal growth soon after birth, with dendritic shortening and atrophy. One possible explanation to reconcile these data is that trisomic 3D environment could be characterized by abnormally low concentrations of trophic factors, or abnormally high concentrations of inhibitory cues that fluctuate during time, differentially affecting dendrites throughout the different phases of their development. In particular, the discrepancy between *in vitro* and *in vivo* data could imply that factors operating in the 3D environment of developing brain, but not in 2D cultures, may be specifically altered by HSA21 trisomy. These factors could consist of missing close contacts between pyramidal neurons and other cell types, such interneurons, astrocytes or other glial cells. The study of trisomic neurons developing within brain organoids (Faundez et al., 2018) may represent a very interesting possibility to further address this phenomenon and to unravel its molecular details.

In contrast with axonogenesis and dendritogenesis, analysis of the late stages of *in vitro* differentiation showed in both models a significant reduction of mature dendritic spines. This result is consistent with the *in vivo* data, supporting the notion that the synaptic defects that characterize DS are at least in part due to cell-autonomous mechanisms, together with additional cell-extrinsic factors that may exacerbate the phenomenon.

From a genetic point of view, the most obvious candidates for such a synaptic effect are the HSA21-located APP and DYRK1A genes. APP is localized to both pre- and post-synaptic boutons, playing a role in the stabilization of the whole synapse. Imaging analyses performed in APP knockout mice showed that layer V cortical neurons exhibited alterations in spine turnover, a reduction in the number of thin spines and an increase in the fraction of mushroom ones (Zou et al., 2016). Another candidate is DYRK1A, because transgenic mice overexpressing this protein show reduced spine density and increased frequency of filopodial spines, similar to DS individuals and mouse models (Martinez de Lagran et al., 2012).

Importantly, our work demonstrated that the spine phenotype observed in the fully described Ts65Dn could be reproduced also in the novel and less characterized Ts2Cje mouse model. For this reason and together with the consistent increase in fertility of both males and females of this line, as compared to Ts65Dn, our results suggest that 2D neuronal cultures of Ts2Cje mice could provide an efficient model not only to identify the genetic factors by which gene dosage imbalance leads to altered synaptic development, but also to screen for pharmacological compounds capable of reverting the phenotype. On this basis, we propose that Ts2Cje should be taken into stronger consideration as a standard model for the study of DS.

## DAta Availability Statement

The raw data supporting the conclusions of this manuscript will be made available by the authors, without undue reservation, to any qualified researcher.

## Ethics Statement

This study was carried out in accordance with the recommendations of Italian Ministry of Health, Istituto Superiore di Sanità. The protocol was approved by the Italian Ministry of Health, Istituto Superiore di Sanità.

## Author Contributions

AC, FD, and GB: conceptualization and experimental design. GB: experiments supervision. AC, MM, GP, FB, and MG: experiments execution. AC, GB, and FD: data analysis. AC and GB: writing – original draft preparation. FD, GB, and AC: writing – review and editing. FD: funding acquisition.

## Conflict of Interest Statement

The authors declare that the research was conducted in the absence of any commercial or financial relationships that could be construed as a potential conflict of interest.

## Supplementary Material

FIGURE S1 **(A)** Percentage of Gad67 positive cells in Euploid and Ts65Dn DIV3 cultures. For euploid 59 cells from 2 mice, for Ts65Dn 67 cells from 3 mice. **(B)** SMI staining of axon at DIV3. Scale bar is 10 μm. **(C)** Brightfield images of DIV5 neurons. Scale bar is 50 μm. Error bars represent SEM. Unpaired two tailed Student’s *t*-test: *p* = 0.71 was considered not significant.
