# Supplementary figures and images for: Neuronal Cell-Intrinsic Defects in Mouse Models of Down Syndrome
Source: Front Neurosci. 2019 Oct 10;13:1081. doi: 10.3389/fnins.2019.01081 (PMC6795679; doi:10.3389/fnins.2019.01081)

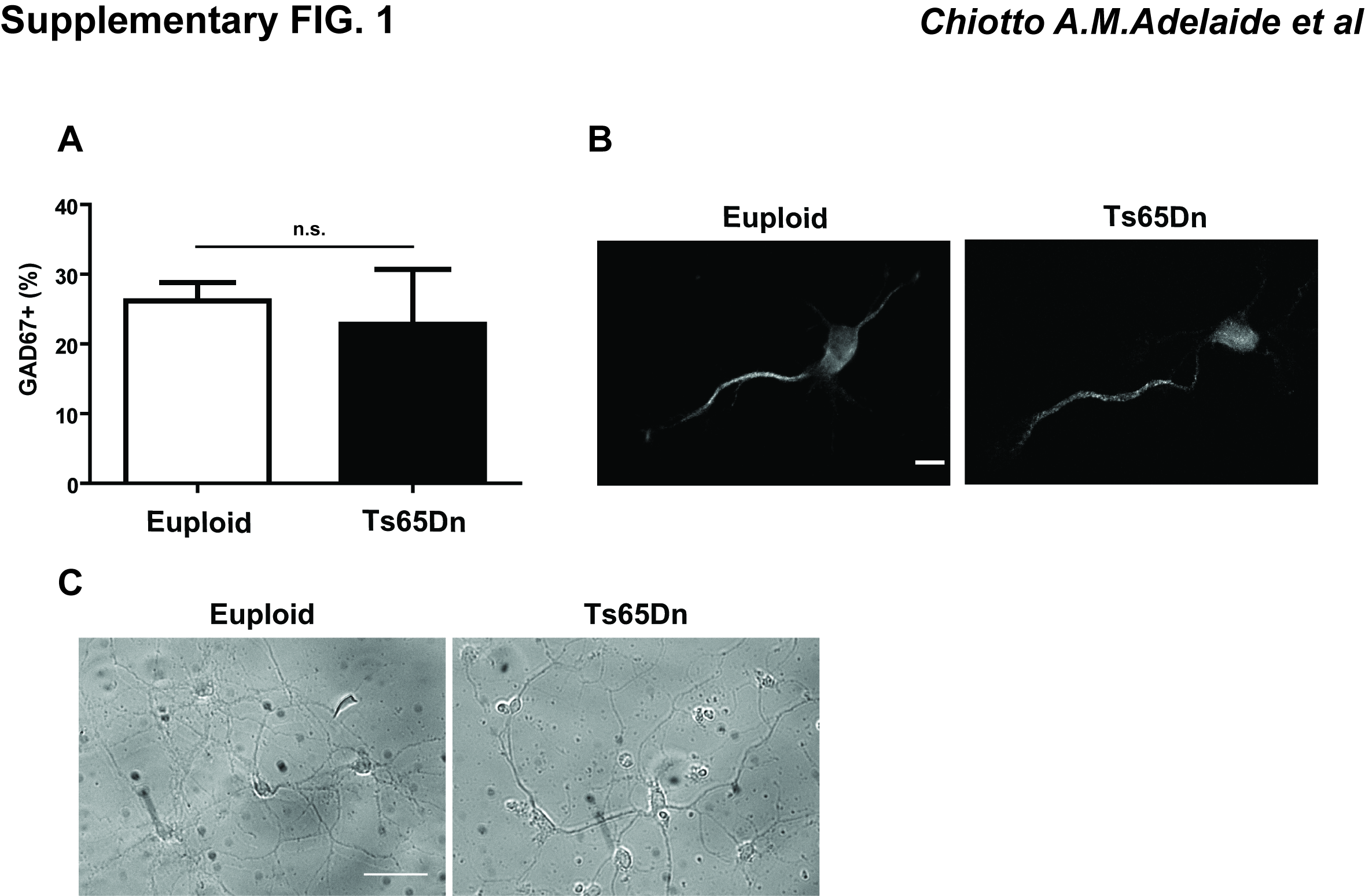

Supplement: FIGURE S1 — (A) Percentage of Gad67 positive cells in Euploid and Ts65Dn DIV3 cultures. For euploid 59 cells from 2 mice, for Ts65Dn 67 cells from 3 mice. (B) SMI staining of axon at DIV3. Scale bar is 10 μm. (C) Brightfield images of DIV5 neurons. Scale bar is 50 μm. Error bars represent SEM. Unpaired two tailed Student’s t-test: p = 0.71 was considered not significant. [file Image_1.tif]
